# Initial researches on neuro-functional status and evolution in chronic ethanol consumers with recent traumatic spinal cord injury

**DOI:** 10.25122/jml-2019-0026

**Published:** 2019

**Authors:** Simona Isabelle Stoica, Ioana Tănase, Vlad Ciobanu, Gelu Onose

**Affiliations:** 1. “Carol Davila” University of Medicine and Pharmacy (UMPCD), Bucharest, Romania; 2. Teaching Emergency Hospital “Bagdasar-Arseni” (TEHBA), Bucharest, Romania; 3. Politehnica University of Bucharest (PUB), Bucharest, Romania

**Keywords:** chronic alcoholism, ethanol, literature review, polyethylene glycol, recent traumatic spinal cord injury

## Abstract

We found differences related to the neuro-functional deficiency and clinical progress, among non-consumers and chronic consumers of ethanol, with recent traumatic spinal cord injury (SCI). We present a synthesis of related data on lesion mechanisms in post-traumatic myelogenous disorders, namely some of the alcohols and their actions on the nervous system, with details on the influences exerted, in such afflictions, by the chronic consumption of ethanol. The subject is not frequently approached – according to a literature review with systematic elements, which we have done before – thus constituting a niche that deserves to be further explored. The applicative component of the article highlights statistical data resulted from a retrospective study regarding the specialized casuistry from the Neuromuscular Recovery Clinic of the “Bagdasar Arseni” Emergency Clinical Hospital, following the comparative analysis of two groups of patients with recent SCI: non-consumers – the control group (n=780) – and chronic ethanol consumers – the study group (n=225) – with the addition of a prospective pilot component. Data processing has been achieved with SPSS 24. The American Spinal Injury Association Impairment Scale (AIS) mean motor scores differ significantly (tests: Mann-Whitney and t) between the control and study group in favor of the second, both at admission (p<0.001) and at discharge (p<0.001). AIS mean sensitive scores differ between the two lots, and also in favor of the study, but statistically significant only at discharge (p=0.048); the difference at admission is not significant (p=0.51) – possibly because of alcoholic-nutritional polyneuropathy. These findings, with numerous related details, later presented in the text, are surprising, which requires further studies and attempts of understanding.

## Introduction

Data on lesion mechanisms in post-traumatic myelin conditions are presented in various papers in the literature – including older ones, written by one of the authors (and contributors) of this article [1] – with details and some new elements, as the research progresses in this area (some minimal general references to this subject are also found in a recent article [2] of the first author of this article). From this perspective, in the introductory part of this journalistic approach, we (re-)emphasize, very concisely, a few important current elements of (morpho-)physiopathology in traumatic spinal cord injury (SCI).

Following spinal cord injury, changes occurred almost instantaneously, constituting *primary lesions*, consecutive, of the nervous tissue: mechanical aggression (compression/traction/torsion) at the cellular level, axonal ruptures – with retractions, dynamically and immuno-molecularly induced (”retraction bulbs” [3] / “retraction clubs at the ends of severed axons” [4]) and expulsion of tissue fragments [5] – but also deterioration of blood vessels from microcirculation of the gray matter [6] with iron release [which contributes to secondary lesions by enhancing the production of toxic free radicals/reactive oxygen species (ROS): Fenton reactions [7, 8] – by the hemorrhages thus produced – including central (hemorrhagic) necrosis [9], central myelin dislocation at the site of the injury [9] including in the region of the ependymal duct – membranal “impact” [10] depolarization of neurons and gliocytes (by abundant release of potassium ions from lacerated cells and inadequate glutamate release: intense excitotoxicity-inducing processes) and local accumulation of dynorphin (with opioid receptor activation – also generating secondary lesions: especially *ischemia* [11]) as well as glutamic acid [12].

It is a known fact – and we already have begun their succinct presentation in this synthetic paradigm – following an injury at the level of the central nervous system (CNS), including at the myelin level, primary and secondary lesions develop [13]. The transition to “the cascade” [14] of secondary lesion events comprises – in summary – the calpain – dependent activation [14, 15], of the NMDA (N-methyl-D-aspartic acid) and AMPA (alpha-amino-3-hydroxy-5-methyl-isoxazole-4-propionic acid) [11] receptors with the consecutive intracytoplasmic accumulation of calcium ions. These, together with the induction, by means of aggressive reaction mechanisms from the category listed above, of an excessive, and therefore unsustainable, a local metabolic augmentation, as a consequence – as important as detrimental – the production, at the mitochondrial level, in marked quantities, of ROS [16, 17], the “oxidative stress” being a real “hallmark” [18] of the redoubtable (secondary) lesional mechanisms of SCI – and not only – which can lead, finally, to insurmountable cellular energetical depletion with the subsequent triggering of the apoptosis mechanisms [19]. The picture of the secondary morpho-/physio-pathological events is amplified including with some vicious cycle developments by triggering of the inflammatory reactions (with consecutive local acidosis), in the complex context of change, in a negative sense, of the local micro ambient: the main cause of the tissular endogenous regenerative trends failure, however, very small and/or improper from the CNS level, in particular of the spinal cord [20]. Damage in the blood vessels and myelin (in the injured area) augments the picture of *secondary lesions*, by local ischemia, intra-neuraxial extravasation of macrophages derived from monocytes with their infiltration into the lesioned myelitic tissue [21] and the occurrence of demyelination/axonal degeneration [11]. At the same time, at the local-regional level (additional “guarantees” of the desolate irreversibility of the whole lesional phenomenon – as the neurons do not reproduce [22]), cellular necrosis occurs immediately, by the destructive mechanical effect of trauma, and by osmolysis subsequent to post-compressive and vasogenic/ischemic edema [23–25] processes of apoptosis, “apoptosis-like” (the latter especially by “protein misfolding” [7, 14]) with the subsequent diminution of the number of neuronal cellular units – interruption or disconnection of intramedullary pathways of communication (extensions of affected perikaryons) and fibrous scarring/gliosis (“reactive astrogliosis” [26], “fibroglial scar” [23]) – all followed by neuro-functional deficit: motor and/or of sensitivity – somatic and/or vegetative (in different degrees) [11] – at sub-lesional level.

Taking into account the individual [27] and social [28, 29] implications of the consequences of the traumatic spinal cord injuries (SCI), various therapeutical modalities were/are under study – of which, in their majority, one of the authors (and contributors) of the current article has [30] already written and published in detail intended to positively influence the clinical (acute/sub-acute/chronic/sub-chronic) progression of these patients – but all, till now, without spectacular curative effects [31].

In parallel, another type of molecule is being researched, potentially having beneficial effects in the treatment, and subsequent post-acute/sub-acute recovery of SCI – polyethylene glycol (PEG). PEG applied to the area of myelogenous injury in adult Guinea pigs produced very good neuro-functional results by having a protective (sealing) effect against damage to the spinal neuron axolemmas of the tested animals [32, 33]. Other studies on Guinea pigs were conducted and showed the benefits in the evolution of lesions after SCI when a PEG single dose subcutaneous injection was administered [34]. PEG would not act as a neutralizer of excitotoxic products in medullary lesions [33], but it would reduce perilesional excitotoxicity by mitochondrial membrane stabilization (after its intra cytosolic entry into the neurons situated in the vicinity of the injured neurons) [33, 35]. PEG applied to extraneuraxial lesioned axons (of the peripheral nerves) would act beneficially for restoring the normal nervous tract by rapidly restoring (after post-lesional PEG application) axonal continuity [34, 36]. The PEG solution works by removing water from cell membranes, which allows them to merge [37].

The use of PEG or a related copolymer with a polymerization index (188) and a molecular weight of 3500 Daltons has been investigated – also in the treatment of several complete myelogenous lesions in larger animals, such as dogs, – and it was found that: “… polymer injection is a safe adjunct to the conventional management of severe neurological injury in dogs. We did not observe any unacceptable clinical response to polymer injection ...” Outcome measures over the 6 to 8-week trial were improved by polymer injection when compared to historical cases [38].

The patients with chronic alcoholism display – as an observation factor resulting from the experience of our casuistry (from the Neuromuscular Recovery Clinic of the Bagdasar Arseni Clinical Hospital) – a progression and a more favorable prognosis of myelin lesions compared to similar non-ethanol consumer patients (see below).

Chronic alcoholism is defined as a long-term exposure (greater than 14–35 days in the case of laboratory animals – rats) [39, 40] at high doses of ethanol. In the case of people, excessive ethanol doses are defined as: “... 8 or more drinks per week for women and 5 or more drinks on any day or 15 or more drinks per week for men ...” [41]), respectively, of more than 8 standard daily ethanolic units (standard drink per day unit = 10 g ethanol) [42]. Taking into account the lack of unitary data on the definition of chronic alcoholism, in the retrospective component we used the diagnostic verbalizations of the doctors (even our own interview experience revealed the positive answers of the interviewed people by their state regarding ethanol consumption as occurring for years). Following the analyzation of literature data, including those on fundamental research elements (specific laboratory techniques related to the approached subject), we extrapolated the calculations regarding the duration of the occurrence of chronic alcoholism in humans at an average duration of about 2.5 years (considering that the equivalence of the life span between man and rat is of 38.4 human days for a rat’s day of life) [43].

Ethyl alcohol affects the development of the human body at all stages, starting with intrauterine life, influencing individuals of all ages, sex, lifestyle or other comorbidities. Numerous studies have been conducted on the negative effects of ethanol abuse on human growth and development, on discernment, on the destructive pathophysiological consequences (at the level of the entire body). During pregnancy, the blood ethanol concentration, at the fetus level, is approximately two third of the mother’s blood [44]. Maternal consumption of ethanol during gestation predisposes the fetus to the risk of developing fetal alcohol syndrome, which may include: diminishing fetal weight, ataxia, intellectual retardation and somatic growth, facial dysmorphism, behavioral disorders [45–47]. Regarding the development of the nervous system in the intrauterine life, the mechanisms by which substances abusively consumed (including ethanol) negatively influence this phenomenon are not clarified, but alcohol is considered to affect normal neurotransmitter–receptor interaction, followed by functional consequences of the neurotransmitters’ actions in adult life [46].

A *physiopathological* neurotoxic ethanolic mechanism can also be encountered in the case of Wernicke encephalopathy (consequence of the thiamin deficiency resulting from malnutrition that occurs in chronic ethanol abuse) [48]. In this case, in addition to the psycho-cognitive disturbances (included in the Korsakoff syndrome), lesions of the intraneuraxial white matter are produced (without being correlated with significant structural changes in the lipids of myelin composition) [48]. The entire histopathological and functional encephalic picture resembles a process of accelerated neural aging [48].

It is also known that peripheral nervous system lesions – chronic alcoholism also being able to produce symmetrical sensory and motor polyneuropathies – can favorably affect the axonal regeneration in the deteriorated central nervous system by IL6 participation, with the activation of the transcription factors cREB (cAMP response element-binding protein) and ATF3 (Cyclic AMP-dependent transcription factor) [1, 49]. Research has also been conducted on the effect of neurotrophin 3 (NT3) in the case of post-traumatic myeloradicular disorders. NT3 is a neurotrophic protein that can improve motoneuronal synaptogenesis which would perhaps explain the discrepancy between the objectified results of the post VMT: much less severe, between the motor scores present at the chronic alcoholics towards the non-chronic consumers of ethanol – distal to a myelo-contusive zone [50,51]. The thorough research on the implications of alcohol abuse shows that NT3 has higher values in males who are chronic alcohol consumers in comparison with patients without the vice of drinking [52].

However, the effects of ethanol are not fully elucidated, and a dynamic connection of glutamate with ethanol intake is also observed [53]. The time required to pass from occasional alcohol consumption to alcohol abuse/ethanolic dependence depends on the individual mental and neuro-structural character (“addiction is a voluntary operant behavior that results from a temporally myopic view of available alternatives in a choice situation”) [53].

In the chronic phase of ethanol dependence, the nervous system decreases its responsiveness to the most important neuro-excitant: glutamate, along with modifications in the conformation of the gamma-aminobutyric acid (GABA) receptors and of the N-methyl-D-aspartate (NMDA) receptors [53]. Due to the decrease in the representation of NMDA receptors in the neural membrane, chronic alcohol consumption causes the excessive production of glutamate in a compensatory way (being a factor that contributes to the morpho-functional changes in ethanol dependence syndrome) [53].

The alcohol molecules are made up of carbon, hydrogen and oxygen atoms bonded through sigma covalent bonds, with the formation of intra-/intermolecular hydrogen electrostatic bonds, where the components are of different mass ratios [54–56]. From the molecular point of view, the ethanol and polyethylene glycol structures are related, (both being part of homologous series of the functional class of the organic substances called alcohols) with several common physio-biochemical properties like hydrosolubility, ability to cross the hemato-neuraxial barrier which is explained in Tables 1–3 of Annex I.

From the physio-pathological point of view, in the recovery after SCI, the astrocytes have a very important role, in (in the acute post-traumatic phase), promoting the formation of perilesional glial scars (starting with the sub-acute phase) [57]. PEG administered after post-SCI favors the functional recovery of the nerves, through the protection of neuronal growth cones and being hindered in its action by neuronal astrocytic perilesional scars [57]. As it is known that alcoholism disturbs the function of astrocytes, a neuroprotective effect of ethanol could be explained in vertebral medullary traumatology [58].

At the same time, a neuroprotective effect of ethanol consumption has also been observed, by inhibiting the formation of harmful ceramide membranes [59]. Ethanol diffuses easily through the neuronal membranes, bonding with both sub-membrane lipid domains and intracellular organisms [60]. In a similar way, it seems that PEG molecules interact with neuronal membranes, providing a neuroprotective effect in the experimental SCI models [61].

Hence, our research endeavor based on clinical and statistical evidence attempts to make further progress by means of applied laboratory tests in our future studies in providing possible explanations of the similarities between these two substances, in terms of their beneficial effects in the case of sub-acute lesions resulted from SCI in chronic ethanol consumers (Tables 1–3 of Annex I).

## Materials, Methods and Results

In relation to the theoretical foundation of this work, with the permission of the Ethical Commision of TEHBA (35517/25.11.2015), we have developed a literature review with systematic elements based on the interrogation of 6 international databases (IDB): Cochrane [67, 68], NCBI/PMC [69], NCBI/Pubmed [70], Elsevier [70], PEDro [71] and ISI Web of Knowledge/Science, respectively in order to determine whether the publications in which we identified potentially qualified articles are ISI indexed [72, 73].

The criteria for the selection of potentially relevant articles consisted in the stratification of the respective research, by means of reference to the time period: January 2000 to February 2019, considering only the works in English. We used the following keywords and keyword combinations for the selection: chronic ethanol consume and spinal cord injury, chronic alcoholism and spinal cord injury, in successive stages of searching. Thus identified 24 articles in NCBI/Pubmed and Elsevier – 1 in the Elsevier database (not ISI indexed) and 23 in the PMC of which 16 ISI indexed (Table 4 of Annex I).

Thus, we have learnt that the target subject of our research is not widely found in the literature. In a brief summary of the tangent problems, there are opinions that the post-VMT recovery process in ethanolic patients is more difficult than in non-alcoholic patients – but not due to reasons linked to the intrinsic neuro-functional reactivity of the spinal cord towards traumatic lesions, but for predominantly contextual reasons, related to the lower cooperation of such patients in the psycho-behavioral aspect, as well as their increased risk of falling, with possible subsequent physical trauma, including spinal cord-injury, in the context of abundant inebriation episodes, with balance disturbances (to which chronic ethanol impregnation of the nervous system can also bring its contribution [47]). In addition, there are studies regarding a possible propensity, for chronic alcoholics with SCI, for neuropathic pain (the list of articles selected by means of the above method can be found in alphabetical order in the appendix).

**Figure 1:**
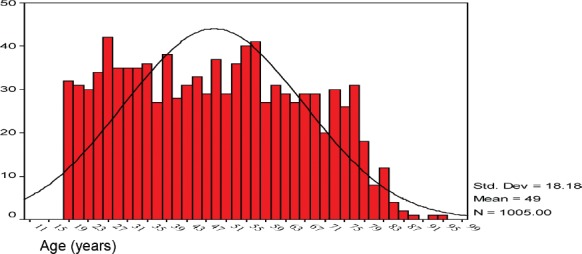
Histogram of patient ages for the lot.

**Figure 2:**
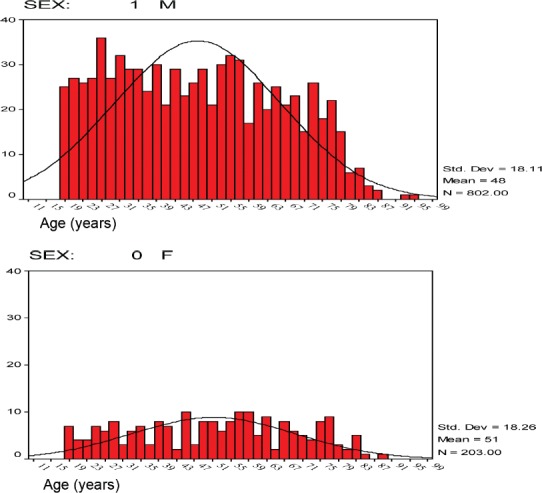
Distribution of patient ages by sex.

In order to objectify the connection between the chronic consumption of ethanol and the (acute/sub-acute) progression of patients with spinal cord injury, we performed a retrospective study of patients admitted in the Neuromuscular Recovery Clinic with SCI diagnosis during the period between January 2005 and August 2018 – a total of 1005 patients of which 196 women, 809 men aged 18–95 years (Figures 1 and 2 of Annex II). To quantitatively objectify the post-SCI neuro-functional deficits for each of the patients analyzed, we applied the AIS standardized assessment scale and the associated (synthetic) Frankel grading in each of the patients analyzed. The AIS (American Spinal Cord Association Impairment Scale) scale quantifies (through scores) the post-SCI motor and sensitivity deficits; while the Frankel scale is very useful for assessing the patient’s functional disorders (using gradations from A to E) [74–76].

For the statistical processing of primary data required for this study, we used the “Statistical Package for Social Sciences” SPSS 24.0 (sequentially accessed in 2018 and 2019) and Microsoft Excel 2010.

In addition to the descriptive statistics elements, there were two stages in the data processing. First, the patients were separated into two groups - ethanol chronic consumers and non-chronic ethanol consumers which are sub-divided into sub-groups, depending on gender, age, environment, causes for the occurrence of SCI, pathophysiological mechanisms of S-C traumatic lesions, myelogenous lesion level, neurological/neuro-dysfunctional level. In the second stage, we performed a comparison through differentiation tests – the patients’ status (chronic ethanol consumers and non-chronic ethanol consumers) and we subsequently performed a comparison of their progress, using the tests: Kolmogogoro-Smirnov, Mann-Whitney/Wilcoxon, chi-square, Fisher exact and Z. The values obtained were statistically significant for different cases where p<0.05. For the specific calculations, confidence intervals (CI) associated with a trustworthiness level of 95% were considered [77, 78].

Given the editorial space, naturally limited, we will present in this article all the ample/exhaustive volume of primary data and consecutive results obtained. The data that we consider to be the most important is the essential data referring to the issues addressed in our study (aspects related to the comparison between the two groups mentioned, the quantified assessments of the neuro-functional deficits and the assessment of the patients).

**Figure 3:**
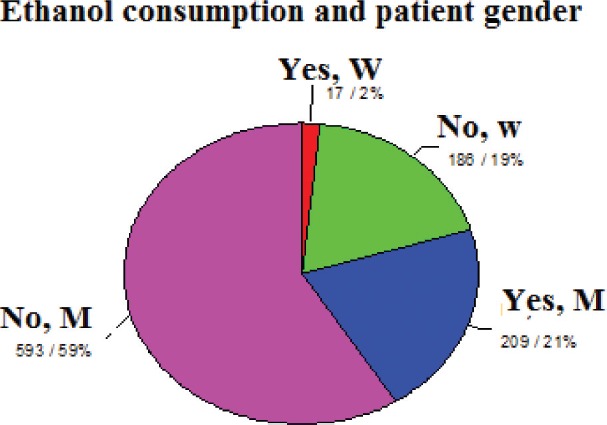
Distribution of patients by gender and alcohol consumption.

Thus of the total patients, 16 women (1.6%) and 209 males (20.8%) were chronic ethanol consumers, and 187 (18.6%) women and 593 men (59%) were not chronic ethanol consumers (Tables 5 and 6 of Annex I, Figure 3 of Annex II).

In Tables 7 and 8 (of Annex I), we can see that the most common neurological levels for the tetraplegic patients are C4 (16.6%), C5 (40.2%) and C6 (20.7%); and for paraplegic patients the neurological levels in most affected SCI are T11 (9.8%), T12 (22.7%), L1 (16.8%).

In Tables 9–11, it can be seen that the pathophysiological mechanisms of spinal cord injury are medullary contusing (19.4%), spinal fracture (55.4%), spinal dislocation (16.4%), spinal fracture and dislocations (8.8%). Of the tetraplegic patients, 181 had medullary contusions; 194 spinal fractures, 139 spinal dislocation, 51 spinal fracture and dislocation. Of the paraplegic patients who were vertebral-medullary traumatized, 14 had medullary contusions, 363 spinal fractures, 26 spinal dislocations, 37 spinal fractures-dislocation.

**Figure 4:**
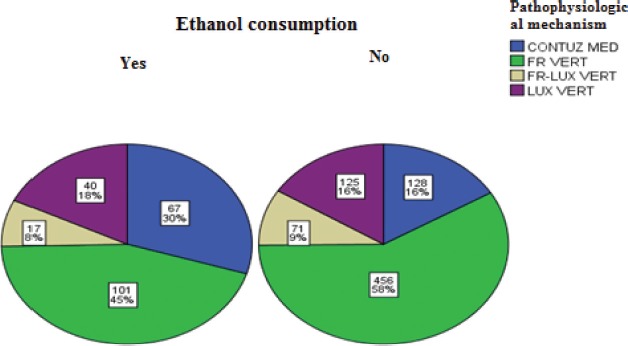
Distribution of patients (chronic ethanolic and non-ethanolic) for the pathophysiological mechanism of traumatic lesion in SCI.

Regarding the physiopathological mechanisms of SCI, (seen in Tables 11 and 12 and Figure 4) the patients with chronic alcohol consumption had the following distribution: 29.8% had medullary contusions, 44.9% spinal fractures, 17.8% spinal dislocations, 7.6% spinal fracture/dislocation. The patients who are not consuming ethanol abusively and chronically presented the following distribution of the physiopathological mechanisms of SCI production: medullary contusion (16.4%), spinal fracture (58.5%), spinal discoloration (16%), and spinal fracture/dislocation (9.1%).

Knowing that AIS motor and sensory scores analytically measure the post-lesional myelogenous neuro-functional deficit, and that the associated Frankel scale grading classifies, synthetically, from A to E, the neuro-functional status, we grouped post-SCI patients – whether or not they were chronic ethanol consumers – a group consisting of patients with Frankel A and B grades (no voluntary somatic movement: complete motor tetra- and paraplegic) and another with C, D, E Frankel grades (with voluntary movements, capable to move, including anti-gravitationally, various somatic segments, especially at the level of the limbs, having muscle force of different intensities, hierarchized on 6 levels); the processing of which is found in Tables 13 and 14. We have found that the patients who are chronic ethanol consumers show results (on the Frankel grading) which are superior to the results of those who do not consume ethanol excessively/chronically. Of the population studied, 166 patients with SCI and chronic ethanol consumers had C, D, E Frankel grades, compared to the 393 non-ethanol consumers, and A and B Frankel grades were found in 59 of the ethanol chronic consumers patients, compared to the 387 cases of non-abusive/chronic ethanol consumers. From a statistical point of view, this finding is supported by the exact Fisher test result (p<0.001) and by the chi-square test (p<0.001), objectifying in patients diagnosed with A and B Frankel grades – a statistically significant difference in favor of a higher percentage of non-abusive/chronic ethanol consumers (49.6% vs. 26.2%). At the same time, in abusive/chronic ethanol consumers, the above mentioned tests revealed a statistically significant difference (p<0.001) in favor of those diagnosed with C, D, E Frankel grades (30.0% vs. 13.2%).

**Figure 5:**
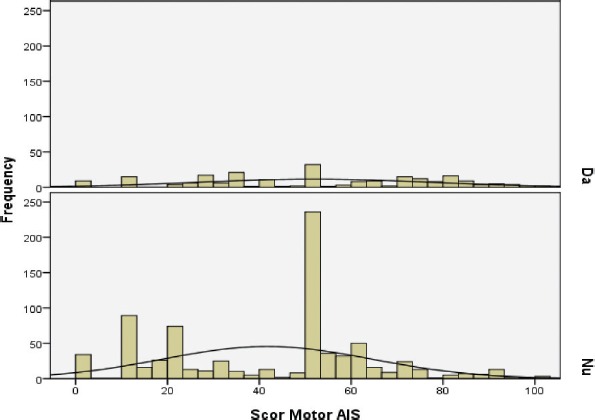
Distribution of AIS motor scores for the chronic ethanolic and non-ethanolic patients.

Table 15 (Annex I) and Figure 5 (Annex II) show that the mean AIS motor scores on admission are higher for patients consuming ethanol in a chronic manner (51.93), compared with non-chronic alcohol consumers (41.7).

We used the Kolmogorov-Smirnov test to verify, from a statistical point of view, the acceptability (or not) of the normality of the data distribution (Tables 16 and 17 in Annex I show the results of the tests performed for the motor AIS score values on admission). From these tables we only take the “final” information, namely the degree of statistical significance: p=0.024 and p<0.001, respectively. In this kind of test, the confirmation of data normality is only accepted if the grade is “high” (over a set threshold in SPSS of 0.20), which is not the case. The unacceptability of data normality does not validate the use of statistical parametric type tests (such as test t). However, the use of non-parametric tests (based on ranks, medians) such as the Mann-Whitney test, remains possible. An example of its application is presented in Tables 18 and 19 (Annex I).

From Table 18 (Annex I) we get information about the highest mean rank which will indicate the “privileged” group, in our case the group of chronic ethanol consumers, and from Table 19 (Annex I) we can only take the degree of statistical significance: p<0.001. In this kind of tests, the confirmation of the statistically significant difference between the groups is only accepted if this degree is “low” (below the conventional threshold of 0.05).

Therefore, the means of the American Spinal Injury Association Impairment Scale (AIS) motor scores differ significantly (Mann-Whitney and t) between the control groups and study groups on admission (p<0.001). The same conclusion is valid for discharge data.

Regarding AIS mean sensitive scores, they differ between the two lots, also in favor of the study lot, but statistically significant only at discharge (p=0.048); the difference at admission is not significant (p=0.519) possibly because of alcoholic-nutritional polyneuropathy “… usually prominent sensory features” [79] (Annex I, Table 20).

Regarding the patients’ progress belonging to the control and study groups – between admission and discharge reflected by the AIS motor scores, respectively AIS sensitivity, there is a statistically significant difference, in favor of the study group (p<0.001, Table 21 in Annex 1). More specifically, the control group has a mean motor score increase of just 1.50 points and the study group has a mean motor score increase of 2.99 points (bigger); regarding the sensitive score, for the control group there is mean increase of 5.76 points less than that for the study group which is a mean increase of 7.69 points. (Annex 1, Table 21).

## Discussion

**Figure 6:**
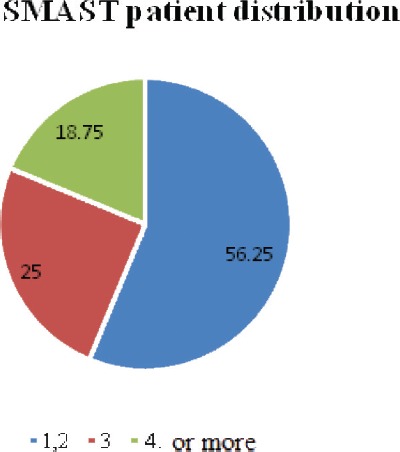
Percentage distribution of SMAST results for patient admitted to the Neuro-muscular Recovery Clinic, at 01.03.2019.

The fact that the patients in the analyzed groups did not have a standardized diagnosis of chronic alcoholism is a relative limitation of this study. After analyzing retrospectively the related clinical medical records, one could not find any such classifications there. That is why we have used, for this purpose, representative definitions from the literature. However, for the sake of rigor we will carry this out following the present research endeavor, in a prospective future extension which carried out on a small pilot sample, the assessment of a number of 16 patients with recent SCI, the discrimination between the state of chronic alcohol consumer and non-chronic consumer, by means of the test used internationally for the assessment of ethanol consumption: Short Michigan Alcohol Screening Test (SMAST) [80, 81]. Thus, we tested patients admitted at the time (on 1 March 2019) in our clinic for recovery after acute/sub-acute spinal cord injury. Specifically, we interviewed 2 women and 14 men. According to the quantification by means of the SMAST questionnaire, the score value 3 is not interpretable as chronic alcoholism, but it is considered “borderline”. Nine patients (2 females and 7 men) got the 1 or 2 score, 4 patients (all men) got the 3 score and the others got a score ≥4; adding the number of patients with on SMAST 3 score to that of non-alcoholic patients, we get a percentage of 81.25% (Figure 6, Annex II). Thus, we find percentage values which are very close to those identified by us in the retrospective study; moreover, the statistical analysis for differentiation shows that the distribution between chronic ethanol consumers and non-chronic consumers (identified using the SMAST quantification) found in the retrospective analysis of the data from the medical records does not differ significantly statistically. This results from the application of the chi-square goodness of fit test: the p-value obtained is 0.829 if the test scores of 3 are not taken into account, and it becomes 0.804, if the patients surveyed are predominantly considered non-chronic ethanol consumers (3 of the 4 patients who got a 3 score on the SMAST testing). These do not contradict the narrative collection for the necessity of the primary data from the retrospective study.

## Conclusions

Altought quite paradoxaly, overall our findings are, we consider, very intersting and worth suplementary research.

## Conflict of Interest

The authors confirm that there are no conflicts of interest.

## Figures and Tables

**Table 1:** Physico- chemical and structural properties of ethanol and PEG [56, 57, 62]

Alcohols/polyols	Ethanol (ethyl alcohol, metilcarbinol, ethyl hydroxyl, ethane monoxide, etiolte, hydroxy ethane)	Poly ethylene glycol (oxide polyethylene oxide, polyoxyetilene)
Method of production	Fermentation of cereals/fruits/some plants	Industrial biosynthesis procedures
Physical properties	Liquid, volatile, flammable, characteristic smell	Molecular weight variables: liquid/solid
Structure	CH3-CH2-OH	H-(O-CH2-CH2)n-OH
Molecular weight	46,076,844 g/mol	18,02-44,05 ng/mol (300–10,000,000 g/mol)
Density	0.7894 g/cm (at 25 °C)	Variable
Boiling point	78.37 °C	182–287 °C
Freezing point	-114,1 °C	Variable
Chemical properties	Water-soluble alcohol	Water-soluble alcohol, methanol, ethanol, acetonitrite, benzene, dichlormetane, acetone. Insoluble in diethy leter , hexane
Biological effects	It crosses the blood-brain barrier, euphoric, sedative, anxiolytic/ anti-depressant, antiseptic, solvent, antitussive, antidote, fuel (7 kcal/g)	Osmotic laxative, coating film for drug molecules, vector in genetic therapy, spinal cord neural lesion fusogen

**Table 2:** Pharmacokinetics of ethanol and PEG (56, 63–66)

Alcohols/ polyols	Ethanol	Poly ethylene glycol (PEG 400/2000/5000)
Absorption	Stomach, small intestine Peck of blood concentration in 30–60 min	Intravenous use
Distribution	Anywhere in the body Rate of extraction=0.2 There are no plasma transport proteins Target: liver, central nervous system, heart	0.07% per gram of neural tissue
Elimination	Limited to the maxiumm 8.5 g/h/70 kg Liver metabolism: *alocoholdehidrogenase, *aldehyddehidrogenase Expiratory (0.7%) – 0.16 L/h Urinary (0.3%) – 0.06 L/h Sweating (0.1%) – 0.02 L/h	PEG 2000 – 1.4 mL/min/kg PEG 5000- 0.4 mL/min/kg

**Table 3:** Pharmacodynamics of ethanol and PEG (35, 50, 54)

Alcohols/polyols	Ethanol	Poly ethylene glycol
Mechanism of action	NMDA receptorantagonist (Decrease of neurotoxicity due to NMDA receptorantagonist) GABA – A receptoragonist 5HT3 agonist Stimulate opiate receptors and production of dopamine	Decreases intracellular release of oxygen free radicals; stabilizes cell membranes against lipid peroxidation; limits injury secondary membrane lesions
Effects	Small doses (0.33 g/kg): improve speed reaction, visual acuity, memory Long exposure(2.0 g/kg): tolerance, dependence, alcoholism Antidote in intoxication with ethylene glycol and methanol Decrease in neurotoxicity due to NMDA receptor antagonism	Merging and repair of injured cell membranes

**Table 4:** Keywords and keywords combination used for search, in BDI, in the review, with systematic elements, afferent, performed, and the quantitative results (articles number of the selection)

Databases	Cochrane	Elsevier		PMC		PubMed	PEDRO	Total	
Keywords	All	All	ISI indexed	All	ISI indexed	All	All	All	ISI indexed
**“chronic ethanol consume” AND “spinal cord injury”**	0	0	0	0	0	0	**0**	**0**	**0**
**“chronic alcoholism” AND “spinal cord injury”**	0	1	0	23	16	0	**0**	**24**	**16**
**Total**	**0**	**1**	**0**	**23**	**16**	**0**	**0**	**24**	**16**

**Table 5:** The main statics indicators of the patients age, for the whole lot

Age (years)	N	Mean	Standard deviation	Minimum	Percentiles	(Median)		Maximum
100	48.56	18,1783	18.04	25	50	75	95.96
5	2			32.73	48.21	63.12	

**Table 6:** Main statistical indicators, by gender

Sexual	N	Mean	Standard deviation	Minimum	Median	Maximum
Feminine	203	51,2306	18,26385	18.04	52,2100	89.32
Masculin	802	47,8862	18,10567	18.08	46,8200	95.96
Total	1005	48,5618	18,17831	18.04	48,2100	95.96

**Table 7:** Neurological level distribution at the studied patients

Neurological level* Diagnosis cross-tabulation				
		Diagnosis		Total
		Paraplegy	Tetraplegy	
Neurological levels	C1	0	8	8
	C2	0	35	35
	C3	0	31	31
	C4	0	94	94
	C5	0	227	227
	C6	0	117	117
	C7	0	41	41
	C8	0	6	6
	T1	0	6	6
	T2	7	0	7
	T3	8	0	8
	T4	31	0	31
	T5	28	0	28
	T6	21	0	21
	T7	18	0	18
	T8	13	0	13
	T9	15	0	15
	T10	34	0	34
	T11	43	0	43
	T12	100	0	100
	L1	74	0	74
	L2	21	0	21
	L3	16	0	16
	L4	7	0	7
	L5	3	0	3
	S1–5	1	0	1
Total		440	565	1005

* The single neurological level is the most “caudal” (the lowest normal) both examined – sensory and motor levels, bilaterally (82).

**Table 8:** Percentage distribution of neurological levels in alcoholic and non-alcoholic patients

Neurological level* Diagnostic cross-tabulation % within diagnostic				
		Diagnostic		Total
		Paraplegy	Tetraplegy	
Neurological levels	C1		1.4%	0.8%
	C2		6.2%	3.5%
	C3		5.5%	3.1%
	C4		16.6%	9.4%
	C5		40.2%	22.6%
	C6		20.7%	11.6%
	C7		7.3%	4.1%
	C8		1.1%	0.6%
	T1		1.1%	0.6%
	T2	1.6%		0.7%
	T3	1.8%		0.8%
	T4	7.0%		3.1%
	T5	6.4%		2.8%
	T6	4.8%		2.1%
	T7	4.1%		1.8%
	T8	3.0%		1.3%
	T9	3.4%		1.5%
	T10	7.7%		3.4%
	T11	9.8%		4.3%
	T12	22.7%		10.0%
	L1	16.8%		7.4%
	L2	4.8%		2.1%
	L3	3.6%		1.6%
	L4	1.6%		0.7%
	L5	0.7%		0.3%
	S1–5	0.2%		0.1%
Total		100.0%	100.0%	100.0%

**Table 9:** Distribution according to the pathophysiological mechanism production of traumatic SCI in all patients

		Frequency	Percent
Valid	Medulary contusion	195	19.4
	Vertebral fracture	557	55.4
	Vertebral fractute and dislocation	88	8.8
	Vertebral Dislocation	165	16.4
	Total	1005	100.0

**Table 10:** Distribution according to the pathophysiological mechanism production depending on the topography of the lesion of traumatic SCI in all patients

Pathophysiological mechanism* Diagnosis cross-tabulation				
Count				
		Diagnosis		Total
		Paraplegy	Tetraplegy	
Pathophysiological mechanism	Medulary contusion	14	181	195
	Vertebral fracture	363	194	557
	Vertebral fractute and dislocation	37	51	88
	Vertebral Dislocation	26	139	165
Total		440	565	1005

**Table 11:** Cross-tabulation according to the pathophysiological mechanism production of traumatic SCI in all patients (chronic alcoholic and non-alcoholic)

Pathophysiological mechanism * Chronic alcoholic and non-alcoholic patients Cross-tabulation				
Count				
		Ethanol consume		Total
		Yes	No	
Pathophysiological mechanism	Medulary contusion	67	128	195
	Vertebral fracture	101	456	557
	Vertebral fractute and dislocation	17	71	88
	Vertebral Dislocation	40	125	165
Total		225	780	1005

**Table 12:** Percentage crosstabulation according to the pathophysiological mechanism production of traumatic SCI in all patients (chronic alcoholic and non-alcoholic)

Pathophysiological mechanism* Chronic Alcoholic and non-alcoholic patients Cross-tabulation				
% Within Consum ethanol				
		Consum ethanol		Total
		Da	Nu	
Pathophysiological mechanism	Medulary contusion	29.8%	16.4%	19.4%
	Vertebral fracture	44.9%	58.5%	55.4%
	Vertebral fractute and dislocation	7.6%	9.1%	8.8%
	Vertebral dislocation	17.8%	16.0%	16.4%
Total		100.0%	100.0%	100.0%

**Table 13:** Frankel grades reparation at chronic ethanolic and non-ethanolic patients

Frankel score (condensed: complete and incomplete deficits) * Chronic Alcoholic and non-alcoholic patients cross-tabulation				
Count				
		Ethanol consume		Total
		Yes	No	
Frankel Scale condensed	A+B	59	387	446
	C+D+E	166	393	559
Total		225	780	1005

**Table 14:** Frankel gradation frequencies in the two main lots of chronic ethanolic and non-ethanolic patients

Chi-square tests					
	Value	Df	Asymptotic significance (2-sided)	Exact Sig. (2-sided)	Exact Sig. (1-sided)
Pearson Chi-square	38.715^a^	1	0.000		
Continuity correction^b^	37.773	1	0.000		
Likelihood ratio	40.314	1	0.000		
Fisher’s exact test				0.000	**0.000**
Linear-by-linear association	38.676	1	0.000		
Number of valid cases	1005				

a 0 cells (0.0%) have expected count less than 5. The minimum expected count is 99.85.

b Computed only for a 2×2 table

**Table 15:** The main statistical indicators of AIS (71) motor scores at chronic ethanolic and non-ethanolic patients

Ethanol consume	N	Mean	Standard deviation	Minimum	Median	Maximum
Da	225	51.93	25,931	0	50.00	100
Nu	780	41.70	22,804	0	50.00	100
Total	1005	43.99	23,910	0	50.00	100

**Table 16:** Statistical processing of motor AIS scores at the admission for chronic alcoholics patients

One-sample Kolmogorov-Smirnov test^c^		
		Score motor AIS la internare
N		225
Normal parameters^a^	Mean	51.93
	Standard deviation	25.931
Most extreme differences	Absolute	0.099
	Positive	0.077
	Negative	–0.099
Kolmogorov-Smirnov Z		1.489
Asymp. Sig. (2-tailed)		**0.024**

a Test distribution is normal

c Ethanol consume=Yes

**Table 17:** Statistical processing of motor AIS scores at the admission for non-alcoholics patients

One-sample Kolmogorov-Smirnov test^c^		
		Score motor AIS la internare
N		780
Normal parameters^a^	41.70	51.93
	22.804	25.931
Most extreme differences	0.224	0.099
	0.114	0.077
	–0.224	–0.099
Kolmogorov-Smirnov Z		6.261
Asymp. Sig. (2-tailed)		**0.000**

a Test distribution is normal.

c Ethanol consume=No.

**Table 18:** AIS motor ranks at admission for the chronic ethanolic and non-ethanolic patients

Ranks				
	Ethanol consume	N	Mean rank	Sum of ranks
AIS motor score at admission	Da	225	588.63	1,32,441.00
	Nu	780	478.30	3,73,074.00
	Total	1005		

**Table 19:** Mann-Whitney test applied to the AIS motor scores at admission of the chronic ethanolic and non-ethanolic patients

Test statistics^a^	
	AIS motor score at addmision
Mann-Whitney U	68,484,000
Wilcoxon W	3,73,074,000
Z	–5,063
Asymp. Sig. (2-tailed)	**0.000**

a Grouping variable: consum ethanol

**Table 20:** Mann-Whitney test applied for AIS mean motor and sensitive scores, admission- discharge of the chronic ethanolic and non-ethanolic patients

Hypothesis Test Summary				
	Null Hypothesis	Test	Sig.	Decision
1	The distribution of Scor Motor AIS extern is the same across categories of Consum etanol	Independent Samples Mann-Whitney U Test	.000	Reject the null hypothesis
2	The distribution of Scor senzitiv AIS intern is the same across categories of Consum etanol	Independent Samples Mann-Whitney U Test	.519	Retain the null hypothesis
3	The distribution of Scor senzitiv AIS extern is the same across categories of Consum etanol	Independent Samples Mann-Whitney U Test	.048	Reject the null hypothesis

Asymptotic signficances are displayed. The significance level is .05

**Table 21:** Mann-Whitney test applied for evolution in admission- discharge of the chronic ethanolic and non-ethanolic patients, reflected by the AIS motor scores AND AIS sensitivity scores

Hypothesis Test Summary				
	Null Hypothesis	Test	Sig.	Decision
1	The distribution of dif scor motor is the same across categories of Consum etanol	Independent Samples Mann-Whitney U Test	.000	Reject the null hypothesis
2	The distribution of dif scor senzitiv is the same across categories of Consum etanol	Independent Samples Mann-Whitney U Test	.000	Reject the null hypothesis

Asymptotic signficances are displayed. The significance level is .05
